# Sex-Dependent Responses in the Retina Proteome to Ocular Hypertension in a Mouse Model of Glaucoma

**DOI:** 10.3390/ijms27167153

**Published:** 2026-08-10

**Authors:** Khadiza Zaman, Autumn B. Morgan, Katalin Prokai-Tatrai, Denise M. Inman, Laszlo Prokai

**Affiliations:** 1 Department of Pharmacology and Neuroscience, College of Biomedical and Translational Sciences, University of North Texas Health Science Center, Fort Worth, TX 76107, USA; khadiza.zaman@unthsc.edu (K.Z.); katalin.prokai@unthsc.edu (K.P.-T.); 2 Department of Pharmaceutical Sciences, System College of Pharmacy, University of North Texas Health Science Center, Fort Worth, TX 76107, USA; autumnmorgan@my.unthsc.edu (A.B.M.); denise.inman@unthsc.edu (D.M.I.)

**Keywords:** glaucoma, mouse, magnetic microbead model, sex as biological variable, retina, label-free proteomics, bioinformatics, pathway analysis, protein interaction networks

## Abstract

Mass spectrometry-based retina proteomics is an emerging method applied to a better understanding of glaucomatous neurodegeneration. This study reports the identification of retinal proteins and processes associated with elevated intraocular pressure using the magnetic microbead model of glaucoma in mice with consideration of sex as a biological variable. The harvested retinas were analyzed by shotgun proteomics via nanoflow liquid chromatography–electrospray ionization tandem mass spectrometry and label-free quantifications. Ingenuity Pathway Analysis^®^ was used for biological interpretation of the resulting dataset. Sex had a statistically significant influence on elevated intraocular pressure-induced changes of retinal protein expressions, and principal component analysis of all identified, verified, and quantified proteins indicated a clear separation of the ocular hypertensive versus normotensive clusters in males, while this separation was far less pronounced in females. Metabolic functions were among the most predominant biological features affected by the increased intraocular pressure. While our results overlapped with many previous findings regarding disease pathology, they have also revealed previously unreported proteins, as well as molecular and physiological functions potentially implicated in glaucomatous neurodegeneration of the retina in a sex-dependent manner.

## 1. Introduction

Glaucoma is the leading cause of irreversible blindness, currently without a cure, and is projected to affect over 100 million people worldwide by 2040 [1,2]. Animal models have been instrumental in studying this disease [3], particularly in advancing our understanding of its detrimental effects on the retina—part of the central nervous system (CNS) and often referred to as the “window to the brain” [4]. Mass spectrometry-based retina proteomics has emerged as a methodology for investigating glaucomatous retinal neurodegeneration, with the potential to address considerable knowledge gaps in the field [5,6]. To survey the impact of ocular hypertension (OHT) as a recognized cause of the disease in humans [1], several early retina proteomics studies were carried out with various rodent models [7,8,9,10].

Recent studies with publicly shared raw data have identified retinal proteins significantly affected by glaucomatous damage from elevated intraocular pressure (IOP) induced via ocular microbead injection [11] and hypertonic saline injections [12] in male rodents. The OHT-independent pathogenesis of glaucoma has also been investigated through identification of affected retinal proteins in an S100B autoimmune model of the disease in male rats [13]. Another retina proteomics study used female rats and cauterization of episcleral veins to increase IOP [14]. Through these investigations, observations of proteome-wide changes and their correlation with disruptions of cellular pathways and networks have offered valuable new insights into the underlying glaucomatous processes [11,12,13], and an animal model of glaucoma with retina proteomics support was also used to advance therapeutic antibody development against retinal neurodegeneration associated with the disease [15].

The importance of sex in glaucoma pathology has been recognized [16,17]. The significance of this biological variable was underscored in animal models of the disease, which included repeated scleroses of episcleral veins or microbead injections into the anterior chamber to induce OHT and subsequent neuroretinal degeneration in rats [18]. Specifically, males showed a higher percentage of retinal nerve fiber and ganglion cell layer thickness losses, as well as worse dark/light-adapted functionality than females. Furthermore, a quantitative proteomics survey has recently revealed sexual dimorphism in the mouse retina [19]. Relying on a magnetic microbead model of glaucoma [20], we present here a new proteomics dataset and results with exploratory bioinformatics analyses, offering the first comparative insights into OHT-induced changes in protein expression across both the male and female glaucomatous mouse retinas on a proteome-wide scale.

## 2. Results

**Figure 1:**
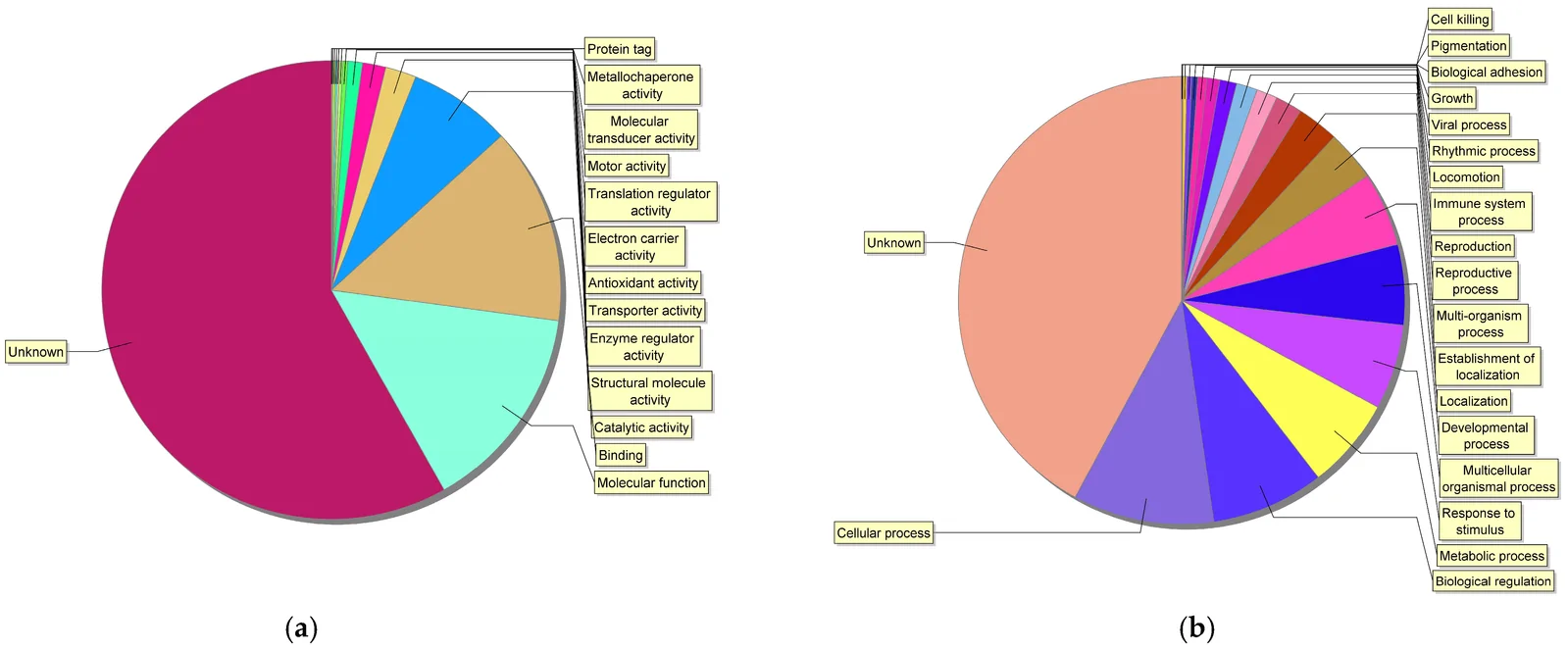
Gene ontology (GO) terms for proteins significantly affected by OHT in the mouse retina: distribution of altered (**a**) molecular functions and (**b**) biological functions. GO annotation selection was performed with an experiment-wide PSEA integrated into the Scaffold DDA software used.

**Figure 2:**
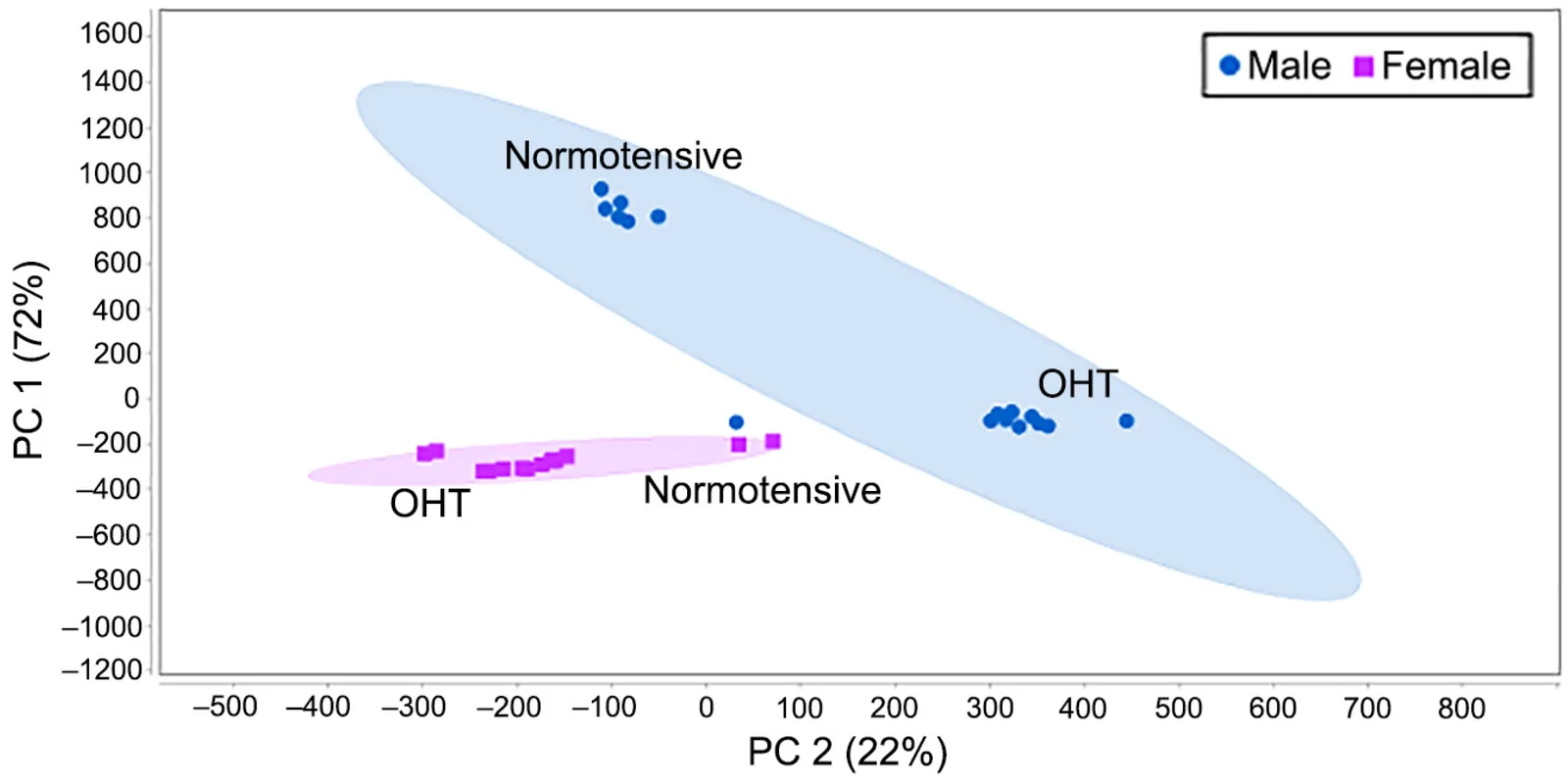
PCA plot of discovery-driven quantitative retina proteomics dataset for the microbead injection model of glaucoma in mice.

Out of over 2000 retina proteins identified and validated using stringent criteria (listed in Table S1 of the Supporting Information), sex × OHT interaction was predominant based on statistical analysis of their expression summarized in Table 1. Proteins significantly affected by OHT in females and males are listed in Table S2a and Table S3a, respectively. Their top molecular and biological functions based on gene ontology (GO) and protein set enrichment analysis (PSEA) are displayed in Figure 1. Overall, statistical analyses of quantitative label-free proteomics (Table 1) indicated sex as a profound biological variable in the mouse microbead injection model of glaucoma, which is illustrated in Figure 2. Glaucomatous and normotensive male retinas also formed clearly separated clusters based on the principal component analysis (PCA) plot from all identified, verified and quantified proteins by Scaffold DDA, while elevated IOP had comparatively moderate shifting from normotensive controls in females in the plot when the results were charted together for both sexes.

**Figure 3:**
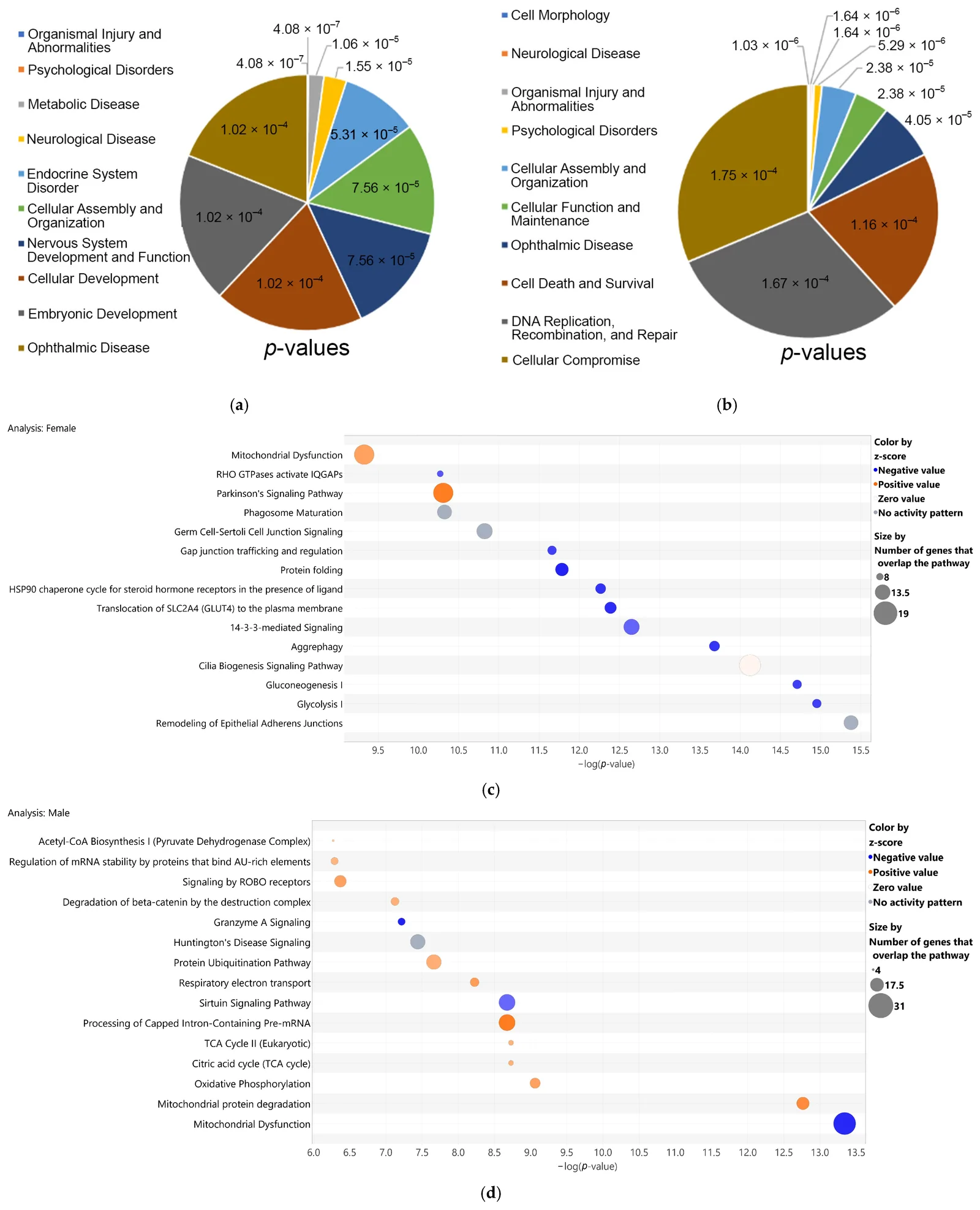
IPA^®^-based bioinformatics analysis of quantitative retinal proteomics results from the microbead injection model of glaucoma in mice: top ten diseases and biological functions significantly regulated by OHT in the (**a**) female and (**b**) male mouse retina. Bubble charts of major canonical pathways triggered by OHT in the (**c**) female and (**d**) male mouse retina.

IPA^®^ associated retina proteins significantly impacted by OHT, based on our results, spanned nearly 50 diseases and physiological functions (Tables S2b and S3b)—with a high predisposition for neurological and ophthalmic disease-specific functions in both sexes. Figure 3 displays the diseases and biological functions returned by the analysis. Specifically, metabolic function, neurological and ophthalmic disease, and endocrine system disorders were among the categories most predominantly affected by OHT in females (Figure 3a), whereas top associations with sustained increase in IOP in males included neurological and ophthalmic disease, along with DNA replication, recombination, and repair (Figure 3b). Out of nearly 200 canonical pathways (Tables S2c and S3c), Figure 3c,d show IPA^®^-generated bubble chart views of the most prominent pathways affected by OHT in female and male mouse retinas, respectively. The most profoundly affected ones according to this analysis were mitochondrial dysfunction, as well as carbohydrate and energy metabolism-related canonical pathways including glycolysis, oxidative phosphorylation and the citric acid cycle. The latter pathways will be further elaborated through additional proteomics studies in a separate follow-up study.

**Figure 4:**
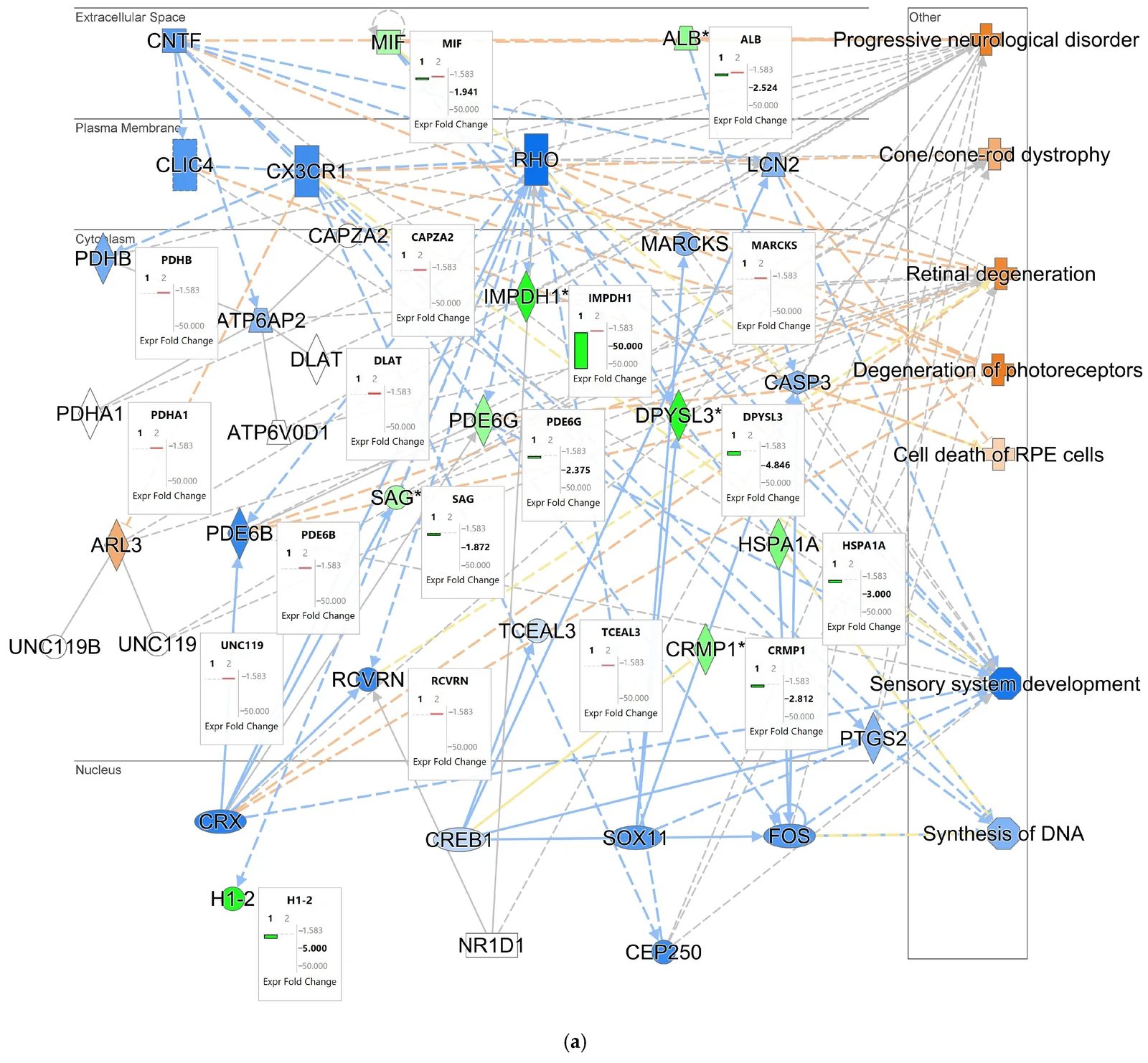

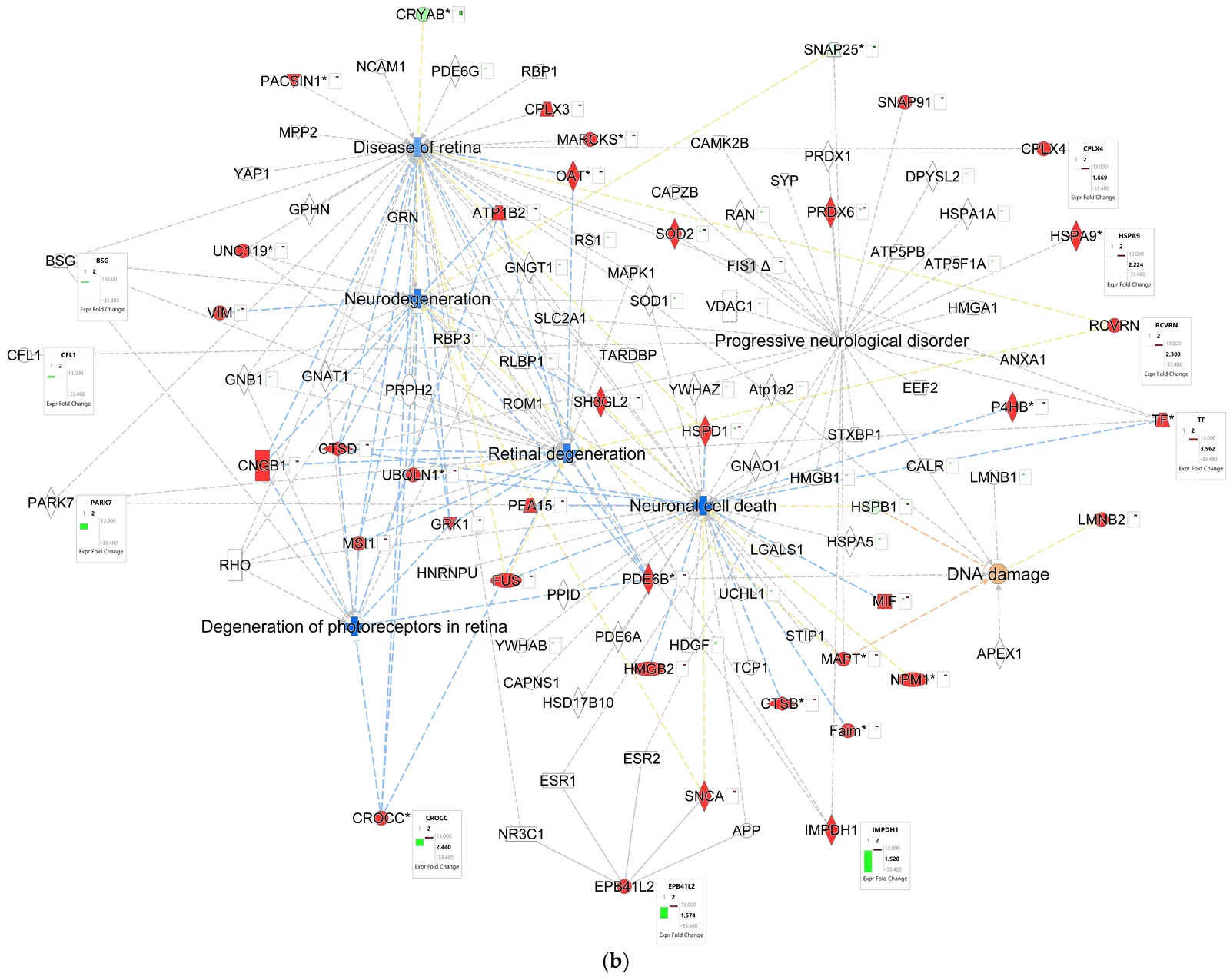
Representative IPA^®^ networks from the retina proteomics dataset for the microbead injection model of glaucoma in mice: (**a**) a protein interaction network linked to development disorder, ophthalmic disease, and organismal injury and abnormalities; (**b**) disease and physiological function network from a subset proteins significantly affected by OHT based on statistical analyses with association to neuronal cell death, degeneration of photoreceptor’s in retina, retinal degeneration, neurodegeneration, disease of retina, DNA damage, progressive neurological disorder according to the software’s knowledge base. Insets: 1st bar—fold change for female OHT compared to female normotensive, 2nd bar—fold change for male OHT compared to male normotensive. Abbreviations of proteins are listed in Table S5, while Table S6 lists spectral counts of CFL1, BSG, PARK7, TF, CPLX4, RCVR, HSPA9, CROCC, EPB41L2 and IMPDH1 for each of the four groups selected for discussion below. Asterisks indicate multiple protein isoforms from the same gene. Red-colored network symbol—upregulation (measured); green-colored network symbol—downregulation (measured); orange-colored network symbol—activation/increase (predicted); blue-colored network symbol—inhibition/increase (predicted); shade of color indicates extent of change in expression, activation/increase or inhibition/decrease. Solid line—direct relationship, dashed line—indirect relationship (blue color: inhibition/decrease, orange color: activation/increase, grey color: activity cannot be predicted, yellow color: finding inconsistent with state of downstream molecule). Arrows indicate direction of activation, causation, expression, localization, membership, modification, molecular cleavage, phosphorylation, regulation of binding, etc. Further details about IPA^®^ legends are available at https://qiagen.my.salesforce-sites.com/KnowledgeBase/articles/Knowledge/Legend (accessed on 30 July 2026).

Several protein interaction networks were also assembled by IPA^®^, as shown by the examples in Figure 4. The 12 protein–protein interaction networks based on retina proteins significantly impacted by OHT in females, males, or both sexes are summarized in Table S4. A top network associated with developmental disorder, ophthalmic disease, and organismal injury and abnormalities is shown using the cellular layout option of the software in Figure 4a, as an example. Importantly, OHT-induced changes in the expression of several proteins within this network were linked specifically to retinal and photoreceptor degeneration, retinal pigment epithelial (RPE) cell death and cone/cone–rod dystrophy. The analysis overlay function of IPA^®^ enabled us to recognize the importance of sex as a biological variable in this regard. For example, a profound decrease in the expression of inosine-5-monophosphate dehydrogenase 1 (IMPDH1) was found in the OHT-female retina, in contrast with the statistically significant increase in the male retina under the same conditions.

As shown in Figure 4b, we also assembled a contextual network encompassing different aspects of glaucomatous retinal neuropathy, including neuronal cell death, photoreceptor degeneration, retinal degeneration, neurodegeneration, retinal disease, DNA damage, and progressive neurological disorder. Based on this network, the analysis overlay function of IPA^®^ enabled our screening for potential disease-associated mechanistic protein marker candidates in a sex-dependent manner. For example, the expression of basigin (BSG), cofilin 1 (CFL1) and Parkinson disease protein 7 homolog (PARK7) was significantly impacted by elevated IOP exclusively in females, while OHT-induced changes significant only in the male mouse retina included complexin 4 (CPLX4), transferrin (TF), recoverin (RCVRN), and stress-70 protein/mortalin (HSPA9). We also noted an opposite regulation pattern of, for example, rootletin (CROCC), Band 4.1-like protein 2 (EPB41L2) and IMPDH1, which were downregulated in females and upregulated in males following OHT.

## 3. Discussion

Relying on a widely accepted model of experimental glaucoma in mice [20], the novel aspect of our study is quantitative retina proteomics focused on glaucomatous neurodegeneration, considering sex as a biological variable in its experimental design. The animals used in this experiment were transgenic mice (GFAP-Cre/ERT2:GLUT1^fl/fl^) on the inbred C57Bl/6 background. The strain is a conditional knockout of GLUT1 in GFAP-expressing mice inducible by tamoxifen administration [21]. In this study, these mice were not given tamoxifen, so on balance they can be considered wildtype with the GLUT1 gene remaining intact. The availability of GFAP-Cre/ERT2 mice and the subsequent suppression of GLUT1 gene transcription with tamoxifen, however, offers an opportunity for designing follow-up studies targeting mechanistic details of OHT-induced glaucomatous retinal neurodegeneration. For our discovery-driven/hypothesis-generating proteomics, the experimental design regarding the number of biological replicates per study group considered the reliance on inbred mice from a small in-house colony, assuring low genetic and environmental diversity [22], as well as the 3Rs (replacement, reduction and refinement) principle of animal research [23].

Previously [11], OHT induced by microbead injection into the eye of male rats was found to decrease the expression of selected retina proteins associated with glutathione metabolism, mitochondrial dysfunction/oxidative phosphorylation, cytoskeleton, and actin filament organization. Conversely, upregulated proteins were involved in the coagulation cascade, apoptosis, oxidative stress, and RNA processing. Overall, the induced glaucoma resulted in differential modulation of nuclear receptor signaling, cellular survival, protein synthesis, transport, and cellular assembly pathways in the male rat retina. While results summarized in Figure 1 and Figure 3 overlapped with many of these previous findings, they have also revealed several previously unreported molecular and physiological functions associated with glaucomatous neurodegeneration of the retina in a sex-dependent manner. In agreement with assessments of two rat models of glaucoma based on retinal nerve fiber and ganglion cell layer thickness, as well as dark/light-adaptation measurements [18], sex also had a statistically significant influence on OHT-induced changes in retinal protein expressions in our mouse model of glaucoma that relied on ocular microbead injections to impede aqueous drainage via the trabecular meshwork (Table 1). In addition, PCA of all identified, verified, and quantified proteins from our dataset indicated a clear separation of the OHT versus normotensive clusters in males, while this separation was far less pronounced in females (Figure 2). Although our new proteomics dataset and exploratory bioinformatics analyses offer comparative insights into OHT-induced changes in protein expression across both the male and female glaucomatous mouse retinas, observations we selected to highlight in the discussion below should be considered in the context of having generated, as examples, testable hypotheses that require follow-up targeted experimental validations regarding both impact on disease pathology and sex differences.

Specifically, IPA^®^ indicated downregulation of glycolysis in female mice, while upregulation of the tricarboxylic acid (TCA) and oxidative phosphorylation were the top canonical pathways related to energy metabolism in the male retina upon IOP elevation (Figure 3c,d). Moreover, the association of OHT with mitochondrial dysfunction was markedly divergent between the two sexes.

Sex differences were also reflected in many protein–protein interaction networks assembled by IPA^®^ from retina proteins significantly impacted by OHT, as illustrated by the top-ranked network presented as an example (Figure 4a). Overall, this protein–protein interaction network was identified as a potential driver of glaucomatous retinal neurodegeneration in the microbead injection model predominantly in female mice. A particularly impacted and contextually important protein in this regard was IMPDH1, the rate-limiting enzyme of de novo guanine nucleotide biosynthesis [24], which was profoundly downregulated by the elevated IOP in the female mouse retina but was affected modestly in males based on our proteomics dataset. Impdh1(−/−) mice develop slowly progressive retinal degeneration with impaired visual transduction despite largely intact photoreceptor structure [25], suggesting that reduced IMPDH1 activity may represent an early mechanistic event in OHT-induced retinal neurodegeneration. Figure 4b’s network highlights additional candidate markers for glaucomatous females. Downregulated BSG has been linked to photoreceptor degeneration and retinal neurotoxicity [26] and to photoreceptor/retinal inflammatory defense [27], though its female-selective effect here is novel. BSG partners with monocarboxylate transporter-1 downregulated prior to degeneration in the glaucomatous optic nerve [28]. PARK7, which supports neuroprotection via oxidative stress relief after retinal/optic nerve injury according to prior studies [29,30,31], was also downregulated by OHT in females. In addition, downregulation of CFL1, a modulator of actin dynamics essential to CNS development [32], may also be a female-restricted effect not previously reported.

Among proteins affected significantly by OHT in the male retina but unaffected in females, upregulated CPLX4 was linked to retinal disease (Figure 4b), with prior work connecting this protein to impaired synaptic transmission signaling in diabetic retinopathy and light-dependent neurotransmitter release [33,34]. Also upregulated only in males were TF (a candidate marker of neuronal death and retinal degeneration, known for neuroprotection in glaucoma via ocular iron maintenance [35,36]), RCVRN (essential for phototransduction [37]), and HSPA9 (mortalin, which mediates axo-protection and modulates mitochondrial dynamics in neurons as a stress sensor [38]). Neither RCVRN nor HSPA9 has previously been linked to glaucoma by proteomics studies, though RCVRN mRNA is reduced in a transgenic open-angle glaucoma model [39]. Our networks (both Figure 4a,b) connect RCVRN to retinal degeneration and, potentially, to glaucoma, motivating further follow-up study to confirm this association.

Consistent with its recent implication in photoreceptor/retinal disease by an optic nerve crush model [40], CROCC was downregulated by OHT in the female retina. However, it was upregulated in males. EPB41L2 showed the same sex-divergent pattern; though lacking direct interactions in Figure 4b, it connected via secondary nodes (APP, ESR1, ESR2, SNCA, NR3C1) to neuronal death, retinal degeneration, and DNA damage. EPB41L2 (protein 4.1G) is required for correct rod synaptic positioning, and its knockdown disrupts retinal neural circuit formation [41], and a related family member has been recently linked to trabecular meshwork actin–cytoskeleton dynamics in a glaucoma model [42].

Altogether, many potential associations captured by the presented proteomics dataset remain to be explored by rigorous investigation of the still incompletely understood glaucomatous retina degeneration, particularly in the context of sex as a biological variable as reflected at the proteome level. Carbohydrate and energy metabolism-related pathways will be further addressed by our additional follow-up orthogonal studies to be published separately, in which we examine protein changes by capillary electrophoresis and Western blots for selected targets, as well as in situ by immunohistochemistry. Since glaucomatous neurodegeneration is complex and multifactorial, a limitation of our reported study is that a single animal model is unlikely to replicate all features of the human disease [3]. Our chosen data-dependent untargeted proteomics methodology is also recognized for undersampling, albeit the alternative data-independent acquisition to increase proteome coverage has disadvantages regarding reliable label-free quantification [43]. Nevertheless, we anticipate that the biomedical information afforded by our dataset (see Data Availability Statement below) will inspire future hypothesis-driven studies focused on the still poorly understood disease pathology of glaucomatous retinal neurodegeneration.

## 4. Materials and Methods

### 4.1. Animals

Mice used in this experiment were generated by crossing GFAP-Cre-ERT2 (B6.Cg-Tg(GFAP-Cre/ERT2)505Fmv/J, Stock #012849) with GLUT1^fl/fl^ (Slc2a1^tm1.1Stma^/AbelJ, Stock #031871) animals from The Jackson Laboratory (Bar Harbor, ME, USA). These mice were part of a larger study examining glucose transporters in astrocytes. The mice used herein carried the GFAP-Cre-ERT2 construct and the flox’d GLUT1 but were not injected with tamoxifen. Hence, there was no Cre-recombinase removal of GLUT1. Male and female (*n* = 4 per study group) mice, 4 months of age, were used. Details of the OHT model were described previously [44,45].

Briefly, paramagnetic polystyrene microspheres (a 1:1 mix of 4 and 8 μm diameter microspheres) were injected into the anterior chamber of mydriatic (0.5% tropicamide), anesthetized (2.5% isoflurane) male and female mice, which were then drawn by a handheld magnet into the iridocorneal angle to impede aqueous drainage via the trabecular meshwork. IOP was measured prior to, and then weekly after, the procedure using a TonoLab rebound tonometer (iCare, Vantaa, Finland). OHT was confirmed by an average IOP increase of 5.8 ± 1.1 mmHg over the four weeks after microsphere injection. The animals were sacrificed via CO_2_ euthanasia followed by cervical dislocation four weeks after the onset of OHT; their eyes were enucleated and dissected for the removal of the retinas. The harvested tissues were flash frozen in liquid nitrogen and stored at −80 °C until they were processed for proteomic analyses.

### 4.2. Sample Preparation

Each collected retina sample was incubated in 200 µL aqueous 8 M urea/25 mM ammonium bicarbonate solution for 60 min to dissolve proteins, followed by centrifugation for 5 min at 1400× *g*. The supernatants were collected, and protein contents were measured by dye-binding assay using a Microplate reader (BioTek Synergy H1:Take3 plates, Agilent, Palo Alto, CA, USA). The samples were aliquoted to contain 100 µg protein each, and the volume was adjusted with 25 mM ammonium bicarbonate for reduction with dithiothreitol (1 mM at 56 °C for 30 min), followed by carbamidomethylation (5 mM iodoacetamide at room temperature and in the dark for 30 min). Subsequently, 8-fold dilutions of the reduced and alkylated sample were made with aqueous 25 mM ammonium bicarbonate solution to dilute the urea to at least <2 M before adding sequencing-grade trypsin (2 µg; Promega, Madison, WI, USA) for overnight digestion at 37 °C. Then, the enzymatic reactions were quenched by adding 5 µL formic acid, after which the solutions were desalted by solid-phase extraction using 1 mL Sep-Pak™ C-18 cartridges (Waters USA, Milford, MA, USA), and the extracts were dried under vacuum (Vacufuge^TM^, Eppendorf AG, Hamburg, Germany) into 1.5 mL centrifuge tubes. The dried residues were dissolved in an aqueous 5% (*v*/*v*) acetonitrile solution containing 0.1% (*v*/*v*) formic acid to obtain 1 µg/µL protein content for the subsequent analyses.

### 4.3. Sample Analysis

The reconstituted samples were analyzed in duplicate by data-dependent nanoflow liquid chromatography–electrospray ionization tandem mass spectrometry (LC–ESI-MS/MS) [46]. Briefly, a LTQ Orbitrap Velos Pro mass spectrometer was connected to an EASY nLC-1000 nanoflow LC system via the EASY nanoelectrospray interface (all equipment was manufactured by Thermo Scientific, San Jose, CA, USA). Separations were performed using a 15 cm × 75 μm i.d. column packed with 2.6 µm BioZen Peptide XB-C18 particles (Phenomenex, Torrance, CA, USA). Gradient elution was performed using water with 0.1% (*v*/*v*) formic acid as solvent A and acetonitrile containing 0.1% (*v*/*v*) formic acid as solvent B after the injection of 3 µL sample solution and 20 min column equilibration at 100% A, while maintaining a constant column pressure at 350 bar. The peptides were eluted at 300 nL/min flow rate using the following gradient program: (i) isocratic at 3% B for 2 min; (ii) linear gradient to 40% B over 60 min and then (iii) 5 min of isocratic at 40% B; (iv) scaled up to 85% B for 2 min; (v) isocratic flow at 85% B for 5 min; and (vi) returning 3% B in 8 min. Positive-ion nanoflow ESI was done by a 7-μm EasySpray emitter (ES 991, Thermo Scientific) at a source voltage of 2.2 kV and ion-transfer tube temperature of 275 °C. Full-scan mass spectra were recorded at nominal mass resolution of 60,000 (at *m*/*z* 400), and 20 data-dependent tandem mass spectra (MS/MS) were acquired in the linear ion trap by collision-induced dissociation (CID) at 35% normalized collision energy and selecting only multiply charged ions (z ≥ 2) for acquisition of MS/MS scans. After a precursor ion was selected for fragmentation, it was dynamically excluded from MS/MS analyses for 60 s.

### 4.4. Data Processing

MS/MS spectra were searched against the UniProt protein sequence database (species: rodent, combined *Mus musculus* and *Rattus norvegicus*, 17,247 and 96,993 entries, respectively) using the SEQUEST search engine [47] (SEQUEST HT) run from Proteome Discoverer (version 2.4; Thermo Fisher Scientific, San Jose, CA, USA) using the target decoy-based method of false-discovery rate (FDR) estimation. Parent ion mass tolerance of 25 ppm, fragment ion mass tolerance of 0.80 Da, and one missed cleavage were used as search filters. Fixed and variable modifications included carbamidomethylation of cysteine and oxidation of methionine, and deamidation of glutamine, respectively. Search results were validated to meet strong criteria by Peptide Prophet [48] and Protein Prophet [49] integrated into the Scaffold 5 software (version 5.3.0; Proteome Software, Portland, OR, USA) including >95% peptide probability, <1% false-discovery rate (FDR) for protein identifications, and at least two identified unique peptides for each protein using the software’s built-in MSFragger algorithm (The Nesvizhskii Lab, Ann Arbor, MI, USA). Label-free quantification relied on the spectral counting method [50] of Scaffold DDA (version 6.5.0; Proteome Software; Portland, OR, USA), with iterative normalization of log_2_-transformed data by Tukey’s Median Polish method and handling missing values through quantile regression imputation of left-censored data (QRILC) algorithm [51]. Statistically significant differences were determined using the software’s two-way analysis of variance (ANOVA) with unpaired *t*-tests for post hoc comparison of protein expression levels between the OHT and normotensive retinas with Benjamini–Hochberg correction for multiple testing. We also used Scaffold DDA’s implementation of PSEA-Quant [52] for protein set enrichment analysis.

### 4.5. Bioinformatics

GO and PCA were performed through Scaffold 5’s (version 5.3.0) and Scaffold DDA’s (Version 6.5.0) built-in bioinformatics tools. To Ingenuity Pathway Analysis^®^ (IPA^®^, Build 9.0, Knowledge Base Content Release of Spring 2026; QIAGEN, Redwood City, CA, USA), we submitted differentially expressed proteins with *p* < 0.05 by ANOVA and at least 50% change in spectral counts upon OHT for annotation and to discover protein interaction networks, as well as their associated canonical pathways, biological functions and diseases based on the software’s curated knowledge base. Overlaps of *p*-values relied on IPA^®^’s calculations based on right-tailed Fisher’s exact tests [53].

## Figures and Tables

**Table 1:** Summary of validated protein identifications and results of statistical analyses.

Statistical Analysis	Total Number of Proteins Tested	Significant ^1^
ANOVA: OHT (main factor)	2200	26
OHT × sex (interaction)		222
*t*-tests: OHT versus normotensive, female	1471	64
*t*-tests: OHT versus normotensive, male	1902	179

^1^ Scaffold DDA (version 6.5.0); *p* < 0.05, corrected for multiple testing (Benjamini–Hochberg).

## Data Availability

The mass spectrometry proteomics data have been deposited to the ProteomeXchange Consortium via the PRIDE [54] partner repository with the dataset identifiers PXD073136 and 10.6019/PXD073136.

## Supplementary Materials

The following supporting information can be downloaded at: https://www.mdpi.com/article/10.3390/ijms27167153/s1.
