# Supplementary material for: Sex-Dependent Responses in the Retina Proteome to Ocular Hypertension in a Mouse Model of Glaucoma
Source: Int J Mol Sci. 2026 Aug 10;27(16):7153. doi: 10.3390/ijms27167153 (PMC13513712; doi:10.3390/ijms27167153)
Supplement: Supplementary file 1 [file ijms-27-07153-s001.zip › ijms-4435321_SupplementaryFigureS9.pdf]

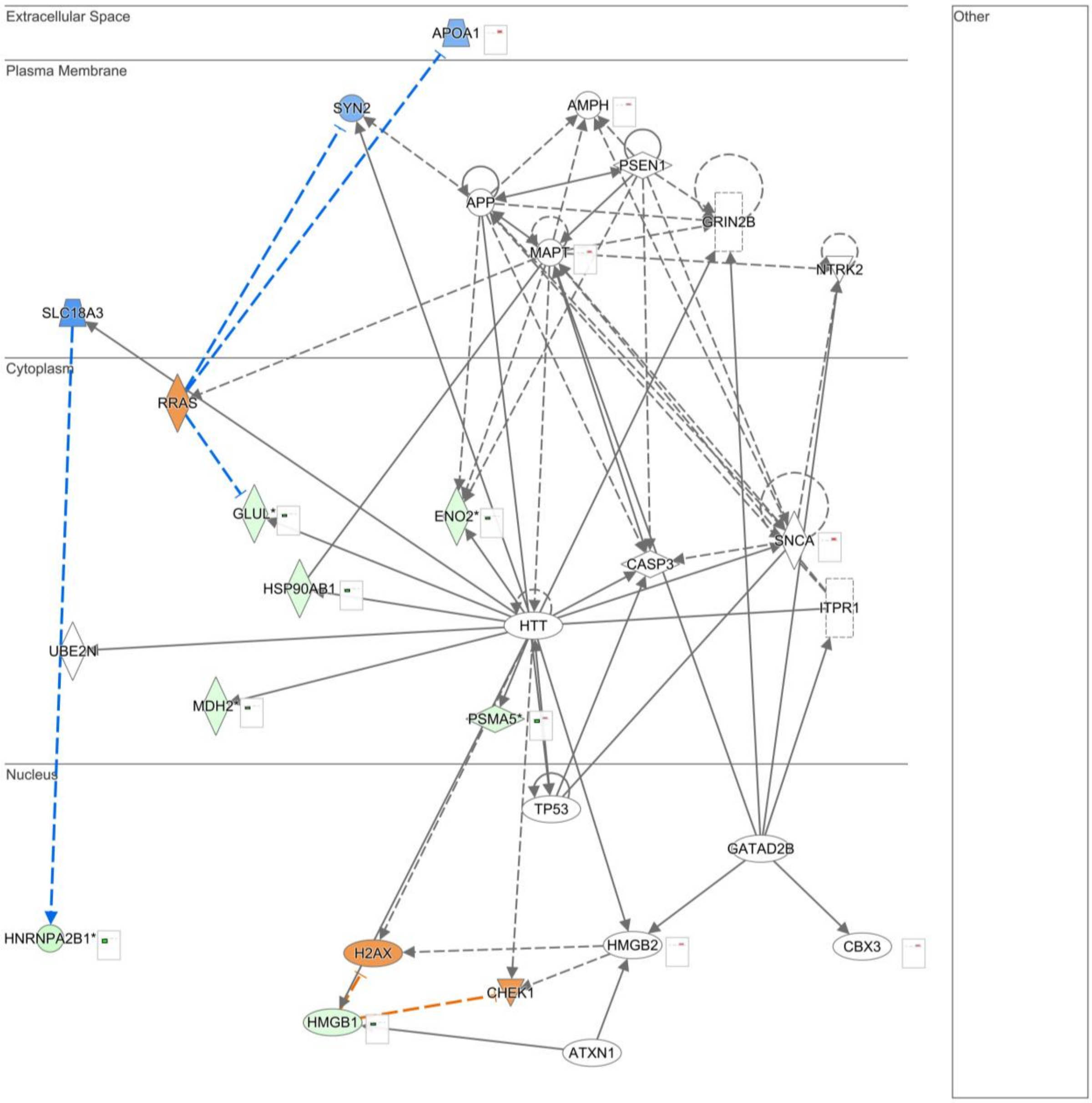

Figure S9. IPA® network linked to cell death and survival, cell morphology, cellular development. The network was assembled from OHT-regulated proteins in the retina of microbead-glaucoma female and male mouse through identification by label-free shotgun proteomics. Red symbols: upregulated, green symbols: downregulated; solid line: direct relationship, dashed line: indirect relationship. Insets: 1st bar: female 2nd bar: male. For further details on symbols and relationships, see https://qiagen.my.salesforce-sites.com/KnowledgeBase/articles/Knowledge/Legend.
